# Understanding complex work using an extension of the resilience CARE model: an ethnographic study

**DOI:** 10.1186/s12913-022-08482-5

**Published:** 2022-09-06

**Authors:** Natalie Sanford, Mary Lavelle, Ola Markiewicz, Gabriel Reedy, Anne Marie Rafferty, Ara Darzi, Janet E. Anderson

**Affiliations:** 1 The Florence Nightingale Faculty of Nursing, Midwifery and Palliative Care, King’s College, James Clerk Maxwell Building 1.32, 57 Waterloo Road, London, SE1 8WA UK; 2 School of Psychology, Queen’s University Belfast, Belfast, UK; 3 NIHR Patient Safety and Translational Research Centre, Imperial College London, London, UK; 4 Centre for Education, Faculty of Life Sciences and Medicine, King’s College London, London, UK; 5 Faculty of Medicine, Nursing and Health Sciences, Monash University, Melbourne, Australia

**Keywords:** Resilient healthcare, Complex systems, Adaptive capacity, CARE model, Work-as-done

## Abstract

**Background:**

Resilient Healthcare research centres on understanding and improving quality and safety in healthcare. The Concepts for Applying Resilience Engineering (CARE) model highlights the relationships between demand, capacity, work-as-done, work-as-imagined, and outcomes, all of which are central aspects of Resilient Healthcare theory. However, detailed descriptions of the nature of misalignments and the mechanisms used to adapt to them are still unknown.

**Objective:**

The objectives were to identify and classify types of misalignments between demand and capacity and types of adaptations that were made in response to misalignments.

**Methods:**

The study involved 88.5 hours of non-participant ethnographic observations in a large, teaching hospital in central London. The wards included in the study were: two surgical wards, an older adult ward, a critical care unit, and the Acute Assessment Unit (AAU), an extension unit created to expedite patient flow out of the Emergency Department. Data were collected via observations of routine clinical work and ethnographic interviews with healthcare professionals during the observations. Field notes were transcribed and thematically analysed using a combined deductive-inductive approach based on the CARE model.

**Results:**

A total of 365 instances of demand-capacity misalignment were identified across the five wards included in the study. Of these, 212 had at least one observed corresponding work adaptation. Misalignments identified include *equipment*, *staffing*, *process*, *communication*, *workflow*, and *space*. Adaptations identified include *process*, *resource redistribution*, and *extra-role performance*. For all misalignment types observed across the five in-patient settings, *process adaptations* were the most frequently used adaptations. The exception to this was for *staffing misalignments*, which were most frequently responded to with *extra-role performance adaptations*. Of the three *process adaptations*, hospital workers most often adapted by changing *how* the process was done.

**Conclusions:**

This study contributes a new version of the CARE model that includes types of misalignments and corresponding adaptations, which can be used to better understand work-as-done. This affords insight into the complexity of the system and how it might be improved by reducing misalignments via work system redesign or by enhancing adaptive capacity.

## Introduction

Resilient Healthcare (RHC) is an emerging field of research that examines safety in healthcare by understanding how work systems adapt to produce acceptable outcomes under variable and fluctuating conditions [1–5]. According to this paradigm, flexible adaptation rather than procedural compliance is essential for ensuring successful outcomes. This differs from many existing safety management practices in healthcare, such as adverse incident analyses, clinical audits, and process control charts, which have emphasised the need to ensure procedural compliance to avoid errors and maximise safety [1, 2, 6–8]. Studying adaptive processes using RHC principles potentially offers insight into how improvement initiatives can increase the number of successful outcomes while reducing the variability of the context, thereby minimising the need for adaptation [9, 10]. However, one barrier to implementing this approach is the difficulty of identifying which aspects of work are relevant for improvement efforts and determining how to structure data collection efforts [10]. In this paper, we explore the challenges faced by healthcare teams in their everyday clinical work and the adaptations they make in response to these challenges. Based on this empirical research, we propose an extension to Anderson et al.’s [9] Concepts for Applying Resilience Engineering (CARE) model that can be used to make sense of challenges and adaptations to better understand flexibility and support work-as-done in complex systems.

To improve healthcare safety, it is important to first understand how healthcare professionals work so that improvement interventions are grounded in the reality of what happens in practice. RHC principles suggest that variability in complex systems like healthcare requires constant adaptation in response to unanticipated conditions to keep the system working, leading to both unsuccessful and successful outcomes [1]. The adjustments made in response to unpredictable variability mean that work-as-imagined (WAI) in protocols and policies and work-as-done (WAD) in practice are different. Furthermore, when the work environment is more stable and has less variability, workers create innovative processes to decrease workload and improve efficiency; this leads to further differences between WAI and WAD [11, 12]. Understanding WAD is necessary to uncover system problems and identify how and why adaptations lead to observed outcomes. Adaptations should therefore be considered a crucial aspect of how work is achieved. Interventions should focus on how to support safe adaptations and maximise the number of successful outcomes [1, 2].

**Fig. 1:**
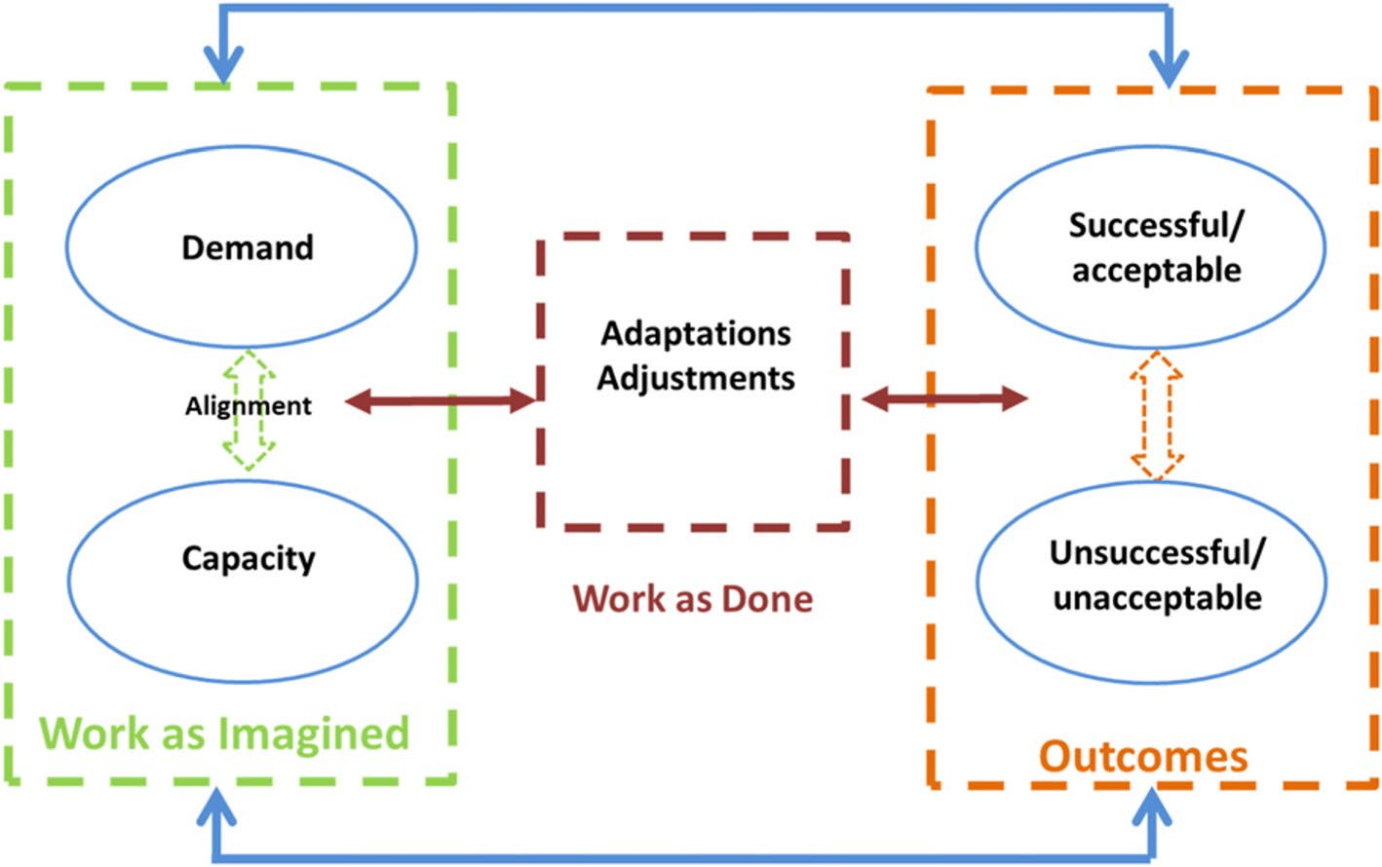
The Resilience CARE Model [9]

The CARE model was developed to illustrate these principles using feedback loops to support research in healthcare systems. The CARE model captures the differences between WAI and WAD using the concepts of ‘alignment’ and ‘misalignment’ [9]. In the model (see Fig. 1), WAI is conceptualised as *alignment* between demand and capacity, where organisations have enough capacity to meet demands*.* Demand and capacity are defined broadly as the system resources (capacity) for delivering the required outcomes (demand). However, in complex systems, there are unforeseeable circumstances, variances, and demands that create *misalignments* between demand and capacity. In these cases, workers must devise ways of working differently to try to meet demand when demand exceeds capacity. These flexible *adaptations* to work are represented by WAD. The model provides a useful means to focus data collection efforts on RHC principles rather than on the myriad aspects of work that can be observed in ethnographic studies. It is thus a conceptual and theoretical focusing device that has been used in conjunction with other methods to guide RHC research in adult and emergency care [1, 13–16]. However, the model does not specify what types of misalignments occur or what types of adaptations are used. RHC research has now developed to the stage where the further specification of the features of resilient healthcare work is needed to guide research and improvement efforts.

Previously, Watt, Jun, and Waterson [17] applied and built upon the CARE model using interviews with blood transfusion staff to identify misalignments (referred to in their paper as triggers) and adaptations. To do this, they interviewed staff about difficulties during blood transfusion and asked how they adjusted their work in response to these difficulties. In their analysis, they deductively applied elements of the Systems Engineering Initiative for Patient Safety (SEIPS) model [18] to categorise the CARE model *misalignments* and *adaptations* according to whether they involved: person(s), tools/technology, task/process, internal environment, or organisation/management. They proposed a new version of the CARE model [9] that incorporates elements from SEIPS. This development is helpful but uses abstract categories to describe misalignments and adaptations rather than direct descriptions of the work. We propose that clearer descriptions of misalignments and adaptations and how they are linked are needed to inform researchers, clinicians, and managers. Accordingly, this study aimed to understand the mechanisms underlying adaptations in clinical teams working in multiple settings in a large, teaching hospital in central London. Specifically, we aimed to identify how clinical work was achieved, including what types of misalignments and adaptations occurred and how misalignments and adaptations were linked.

## Methods

We undertook 88.5 hours of non-participant ethnographic observations in a large teaching hospital in central London. Purposive sampling was used to select five diverse ward areas to represent multiple in-patient settings within the hospital. The included ward areas had teams that differed according to goals, membership, longevity, and type of tasks to better represent a spectrum of teams in the in-patient setting. The wards included in the study were: two surgical wards, an older adult ward, a critical care unit, and the Acute Assessment Unit (AAU), an extension unit created to expedite patient flow out of the Emergency Department. Preliminary results from this analysis have been reported in a conference paper [12] and a separate analysis related to hospital pressures has also been published [19].

### Data collection and analysis

As reported in Sanford et al. (2022), the research team consisted of clinicians and non-clinicians from varied backgrounds, including social science, psychology, human factors, nursing, medicine, and education [19]. Non-participant ethnographic observations were conducted between October 2018 and March 2019 by two members of the research team (ML and OM). The researchers were both trained social scientists familiar with RHC and the CARE model. Events were observed where they occurred naturally, and so observations were not necessarily confined to the ward area. The observations included activities such as: ward rounds, medication rounds, and general ward activities, as well as coordinating events such as handovers, board rounds, multi-disciplinary team (MDT) meetings, and bed meetings [12, 19]. In-depth ethnographic field notes were transcribed and uploaded into NVivo 12 for storage, organisation, and analysis on a password-protected computer. Identifying information was removed.

A combined deductive-inductive approach to thematic analysis was used and was carried out in three iterative phases between July and October 2020. In phase one, after initial data immersion, deductive codes based on the resilience CARE model were applied [9, 12]. The transcripts were coded by author NS to identify segments of text that demonstrated instances of “misalignment” and “adaptation.” In line with the CARE model, misalignments were defined as an element of the organisational context that resulted in demand exceeding capacity. Instances of misalignment were initially identified in the transcripts based on descriptions of capacity issues; conversations among staff members about challenges; observations of resourcing issues; and discussions among staff members about addressing demands. Adaptations were defined as any adjustments made to work in response to to increased demand that exceeded capacity. Adaptations were identified in the transcripts based on descriptions of workarounds and adjustments; staff conversations about overcoming challenges and increasing capacity; and observations of workflows that circumvented formal processes. These two deductive themes were discussed and iterated with the larger research team during team meetings to ensure conceptual agreement was present throughout the analysis process. The team met regularly to agree and interpret results, which involved reviewing the data and sense-checking the coding. One member of the research team was a physician and two were nurses. In addition to clarifying observed events with participants during the ethnography, the research team members’ familiarity with the clinical environment was essential to understanding and identifying the phenomena of interest during the analysis [19].

Next, in phase two, a round of inductive coding was completed by NS to identify relevant sub-codes. The sub-codes were constructed to sort text coded for misalignments and adaptations into sub-categories based on situational characteristics and commonalities. This analysis identified six types of misalignments: equipment, staffing, communication, space, process, and workflow, and three primary types of adaptations: extra-role performance, resource redistribution (sub-sub-categories: staff and equipment), and process (sub-sub-categories: who, how, and when). These categories are presented and discussed in detail in the results section of the paper. The team also met regularly during the inductive phase of analysis to agree and interpret results, which involved reviewing the data and sense-checking the coding and developing framework. Once the sub-codes had been finalised, 25% of the transcripts were independently coded by a second researcher (OM). Disagreements were discussed, sub-code descriptions were clarified, and coding was amended. The final agreement between coders for the aggregated top-level codes of misalignment and adaptation was 92.60 and 95.23% respectively, indicating that the coding framework was highly reliable.

In the third and final phase of data analysis, the relationship between misalignments and adaptations was examined to determine how misalignments were linked to adaptive actions. A paired, matrix coding query was run in NVivo 12 by NS to identify segments of text which had coding for a misalignment followed by the corresponding adaptation. The matrix coding query function is a search function and identifies text coded as both a misalignment and an adaptation. The type and number of adaptations for each misalignment were quantified. This allowed us to determine and quantify when and how often specific misalignments were responded to with specific adaptations. These excerpts were reviewed in detail by NS and ML, who synthesised these scenarios in a table. The synthesised data were then reviewed by the entire team during team meetings for further discussion and interpretation.

### Ethics

As previously reported, the study had ethical approval (REC REF:18/WA/0218) and formal approval from Trust leads [12, 19]. In each of the participating ward areas, the lead doctor(s) (e.g., consultant) and the lead nurse(s) (e.g., ward matron) were provided with information about the study in the form of a verbal presentation and written study information sheets. For a ward to participate in the study, written informed consent was required from all clinical leads on that ward. Staff leads also provided their consent for researchers to shadow them during their routine work. In total, 36 healthcare staff provided written informed consent to participate, enabling data collection of routine work across five ward areas [12, 19].

## Results

In this section, we first present an extension of the CARE model (CARE Model 2.0), which includes the types of misalignments and adaptations identified, followed by results illustrating pairings of misalignments and adaptations. Finally, we present qualitative data to illustrate the nature of the identified misalignments and adaptations.

There were 365 misalignments between demand and capacity observed of which 212 could be linked to an adaptation. Misalignments were grouped into six categories: *equipment, staffing, communication, space, process, *and *workflow. *Adaptations were grouped into three categories: *extra-role performance, resource redistribution, *and *process.* *Process adaptations* were further divided into the subcategories of *who*, *how*, and *when* to indicate in what ways processes were adapted. *Resource redistribution adaptation* was divided into the categories of *equipment *and *staff *to indicate which resources were redistributed. Table 1 provides an overview of the CARE Model 2.0 framework, which builds on Anderson et al.’s [9] model by contributing empirically identified misalignment and adaptation types with examples from our observations.

**Table 1:** CARE Model 2.0: Types of Misalignment and Adaptation with Descriptions and Examples from our Observations

**Misalignment Type**		**Description**	**Observed Example**
** Equipment**		Equipment shortage and/or equipment is broken, not fit for purpose, or faulty	There are not enough computers on the ward
** Staffing**		Unexpected shortage of staff and/or staff skills mix does not fit needs	A staff member calls in sick, leaving the ward short-staffed
** Communication**		Message is not accurate, clear, and/or consistent	The medical team is not informed that the consultant has been reassigned to a different team
** Space**		Space is poorly designed for purpose	The team meeting takes place in a hallway, lacking privacy and blocking traffic flow through ward
** Process**		Process is not efficient, reliable, easy, coordinated, and/or achievable	The patient is medically ready for hospital discharge. It is unsafe for them to discharge home independently, but they do not qualify for an increased package of care
** Workflow**		Staff are taken away from the task they are working on to tend to another task	A nurse providing patient care is interrupted with a question from another team member
**Adaptation Type**		**Description**	**Observed Example**
** Extra-role Performance**		An individual spontaneously assisting with work that is not their direct responsibility	The ward manager delivers meal trays to help when the ward is busy
** Resource Redistribution**	*Equipment*	Redistributing equipment to areas of greater need	The nurse borrows a glucometer from a neighbouring ward
	*Staff*	Redistributing staff to areas of greater need	The ward borrows an additional nurse from a neighbouring ward when they are short staffed
** Process**	*Who*	Changing who does a process by reshuffling tasks among team members	The consultant leaves to attend to an urgent situation on another ward. The junior doctor and nurse practitioner divide up and complete the outstanding tasks while she is away
	*How*	Changing how a process is done	The computer is not working, so the doctor writes paper notes instead
	*When*	Changing when a process is done	The patient is not available when the nurse tries to give them their medications, so the medications are given later

Next, misalignments were paired with adaptations, which enabled us to capture how frequently teams utilised each adaptation type in response to each misalignment type (see Table 2). The heat map below illustrates that for all misalignment types, except *staffing misalignments, process adaptations* were the most frequently used. *Process adaptations* were most often implemented by adapting *how* a process was done. *Extra-role performance adaptations* were used most frequently in response to *staffing misalignments* and were the second most common adaptation type overall after *how process adaptations.*

**Table 2:**
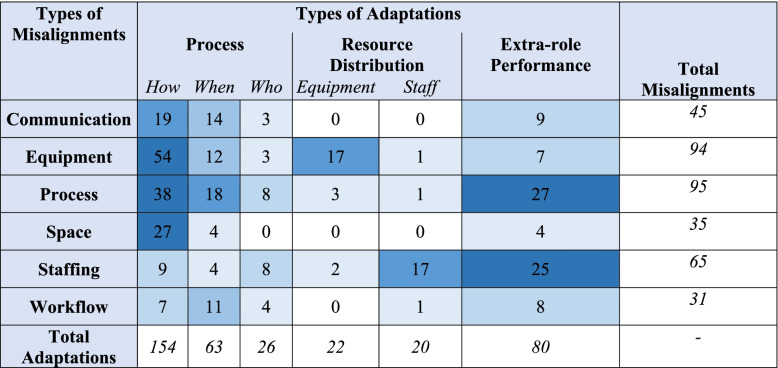
Number of Adaptations by Misalignment Type

KEY:  0  1–5  6–10  11–15  16–20  20+

Some adaptations had natural pairings with specific misalignments. For instance, the *staff redistribution adaptation* was almost exclusively utilised in response to *staffing misalignments*:

At this stage [the second-year doctor] leaves the ward round to join the team who needed the extra help so she will not be able to work with this team for the rest of the day. (Field Notes from Ward Type 1, Observation 11)

Equally, the *equipment redistribution adaptation* was almost exclusively utilised in response to *equipment misalignments:*

A porter comes to take another patient for an investigation. The nurse explains that the patient will need to go with oxygen, but that this ward does not have any oxygen cylinders so [they] suggest the porter goes to XX ward to get one for the patient. (Field Notes from Ward Type 3, Observation 19)

These staff and equipment adaptations were rarely linked with other misalignment types. However, both misalignment types were also linked with other types of adaptation, namely *process* and *extra-role performance adaptations*. In these cases, in our observations, *resource redistribution adaptations* were usually requested by team members as a first-line adaptation. For instance, team leaders requested bank staff, visited neighbouring wards to plead for extra staff members or equipment, or requested these resources through another formal pathway. When the request was not fulfilled (e.g., *resource redistribution* did not take place) this necessitated that staff use a different adaptation, for example, a *how process adaptation*:

The nurse calls the nurse in charge (NIC)…to ask what time someone can relieve her for lunch…the nurse explains to me that [the] ward [is] very busy and there isn’t anyone to relieve her…so… [the] NIC suggested that she leave the [health care assistant] on the ward and have her lunch in the discharge lounge next door so she is easily accessible in the event of any problems. (Field Notes from Ward Type 3, Observation 19)

*Process adaptations* were the most used adaptation and were used in response to a variety of misalignments, including *space misalignments:*

…The meeting room usually used for this meeting is being refurbished and has been out of action for three weeks…They can’t have the meeting in the doctors’ office because it has windows onto the main corridor which are open so patients/public can hear conversations, so it was decided to do the meetings in the corridor. (Field Notes from Ward Type 1, Observation 13)

In this case, in the absence of adequate space, the team used a *how process adaptation,* changing how they ran the meeting by changing the location.

Although *process adaptations* were also used to adapt to *process misalignments*, which was another natural pairing, hospital teams also adapted to *process misalignments* by taking on additional work that was outside of their responsibility using *extra-role performance adaptations*:

I follow [the] Ward Manager (WM) back to [the] ward. She complains that she has a lot to get done, but has constant distractions... She says [the] switchboard…put all external telephone calls through to [her ward], as many patients will at least have passed through there. This takes up a lot of time…as she has relatives calling to ask where patients have been moved to…[This] requires finding and logging onto a computer… and looking up a patient’s records. She says even more annoying is when other departments (e.g. radiology or endoscopy) call up to do the same, when they could just…look it up themselves… (Field Notes from Ward Type 1, Observation 13)

In other instances, teams were able to shuffle tasks among team members to avoid disruption when faced with misalignments, using *who process adaptations.* For example, in response to a *workflow misalignment,* the Advanced Nurse Practitioner (ANP) took over documentation from the first-year doctor when the doctor was called away:

The [doctor] gets called on her…mobile and leaves the patient’s bedside to take the call. The ANP who has re-joined the ward round takes over the documentation of the review. (Field Notes from Ward Type 4, Observation 23)

*How* and *when process adaptations* were utilised more frequently than *who* process adaptations. Interestingly, individuals also took on work that was not their direct responsibility (*extra-role performance adaptation*) more often than teams responded by reshuffling tasks among the team (*who process adaptation*).

In some cases, multiple adaptations were used in a response to a single misalignment, such as changing both *how* and *when* a process was done:

On this ward, there are no available [computers] so the [doctor] writes notes on her handover sheet which she will have to transcribe later… [this creates a] delay in communication for those relying on ward round notes to know what’s going on with the patient... while the [senior doctor] is seeing the patient, the lack of computers means [they] can’t check patient observations during [the] review… he delegates this to the first-year doctor to [do later]… (Field Notes from Ward Type 4, Observation 22)

In this scenario, an *equipment misalignment* prompted *how* (writing paper notes) and *when* (transcribing the paper notes in the electronic medical record and checking the patient’s observations later) *process adaptations.*

In some observations, a domino effect of a series of misalignments and adaptations was visible:

[The] nurse shows the patient [the] discharge summary and goes through all the written content…Patient notices an error on discharge summary – [the] wrong date [is] written for an outpatient investigation…[the] patient appears well informed and clear with…plan, does not seem fazed by error on summary… Summary will not be updated/corrected prior to patient’s discharge…doctors are encouraged to write discharge summaries as early as possible, but plans may change, meaning that patients either go home with wrong or vague discharge summary, which is also sent to [primary care physician], or [they] are sent multiple versions of the summary. (Field Notes from Ward Type 4, Observation 17)

In this case, a *process adaptation* utilised by the medical team (writing the discharge summary early) led to a *communication misalignment* (the date for the outpatient investigation is communicated incorrectly on the discharge summary to the patient and the primary care physician). The adaptation was that the nurse and patient discussed the error to ensure understanding of the plan rather than updating the discharge note (*how process adaptation).* It is unclear if the correct information is ever communicated to the primary care physician and what the impacts of this *communication misalignment* are, if any.

## Discussion

Misalignments and adaptations are ubiquitous features of how clinical work proceeds. As RHC principles and this study suggest, clinical care is a process of constant adjustments in response to the conditions of the work system. These adaptations are a key feature of work regardless of the outcome and are not unique to situations in which things go wrong. On the contrary, workers are adept at devising adaptations to ensure the system keeps working when misalignments occur. These results echo the evidence base on the technical work of healthcare that has similarly found that workers need to adapt to compensate for poor system design [20–24] and these results provide more detail about how adaptations are provoked and implemented. In our data, process adaptations were implemented the most frequently and most often workers changed *how* processes were done in response to misalignments.

Clinical work is not linear, so it is not possible to completely specify work-as-done. However, utilising a common vocabulary to uncover, to the best of our ability, how work takes place allows us to consider the actions required for work to continue. Understanding how misalignments provoke adaptations and what these imply about how the system functions is important for designing effective improvement efforts [9, 25]. Misalignments and adaptations, regardless of type, have implications for the quality and safety of patient care and could result in both positive and negative potential impacts. From our observations, it was clear that outcomes and impacts were often subtle and had both positive and negative effects which occurred over different time scales and for different system actors. For example, writing notes by hand as a *how* process adaptation during an *equipment misalignment* was a successful adaptation and outcome because ward rounds were able to continue. But several visible negative impacts should be considered when determining the appropriateness of the adaptation. For instance, the physician had to duplicate efforts by transcribing and publishing the note on the computer later in the shift, there was a delay in communication to the wider team as a result, and the ward round team could not view the patient’s scans during the round. Identifying these subtle effects that occur at different times and locations for different actors is challenging, but further research should focus on better defining the outcomes and impacts of different adaptations, understanding what constitutes a successful outcome for whom, and determining how to support effective adaptations.

The strength of our analysis is that identifying and pairing misalignments and adaptations allows us to consider the trajectory of actions required for successful patient care. This affords insight into the complexity of the system and how it might be improved through reducing misalignments (work system redesign) or by enhancing adaptive capacity [9]. Various experts have called for greater focus on system design for improving the quality of care [26, 27]. The CARE Model 2.0 provides an easily adoptable language that can be used to categorise misalignments and adaptations. Studying misalignments using this framework will afford greater insight into aspects of the work system that could be improved by better-designed systems and processes. We also highlight the limits of adaptation to compensate for system design problems. For example, staffing levels cannot always be increased, so other actions are needed so the system can still work if there are staffing shortages.

There are several strengths of this study and its resulting framework. First, there is some conceptual overlap between our findings and other models, such as SEIPS 2.0 [18], SEIPS 3.0 [28], SEIPS 101 [29], and Watt et al.’s [17] enhanced CARE model, which combines ideas from SEIPS 2.0 [18] and the CARE model [9]. The conceptual consistency between the new CARE model 2.0 and others strengthens our confidence in the results. That said, we consolidate and clarify some of these previously identified themes. For example, both Holden et al. [18] and Watt, Jun, and Waterson [17] differentiate between IT and non-IT equipment. Given the ubiquity of digital devices and interfaces, we decided to consolidate these two categories [17]. Holden et al. [18] also break processes into “small tasks” and “large processes.” We combine these themes into one category entitled ‘process misalignment.’ In our data, sometimes a misalignment took place during a ‘small task’ within a ‘large process,’ which makes distinguishing these impractical. In addition to these consolidations and clarifications, we also contribute new findings, such as the expansion of *process adaptations *into three sub-categories of *who, how,* and *when,* which provides useful insight into in what ways a process is adapted. Our framework also differs in its identification of new misalignments such as *space, communication, workflow,* and *staffing* as well as *extra role performance* and *resource redistribution adaptations*. Second, a strength of our framework compared to previous ones is our use of descriptive labels for the types of adaptations. We wanted the categories to directly describe the actions involved rather than reflecting abstract aspects of the work system, such as tasks or equipment, or technical terms such as ‘enabling conditions.’ Such abstract descriptors require further interpretation to understand the activities they describe. Our results confirm and extend previous work in this area and provide a framework for describing WAD. Third, the framework was empirically developed using data from multiple healthcare settings, teams, and tasks, strengthening its validity and reliability. To our knowledge, it is the first to identify how misalignments and adaptations are linked by tracing trajectories of action in routine clinical work. Fourth, the study was conducted by a multidisciplinary team of experts from nursing, medicine, psychology, and human factors. Our varied backgrounds have afforded greater insight into the complexities of clinical care, misalignments, and adaptations.

There are several possible limitations of this study and the resulting framework. Firstly, there may be factors besides misalignments, such as personal preference, that spark adaptations that were not immediately observable and have not been accounted for yet in this framework. Additional research is needed to explore other factors, besides misalignments, that could precipitate adaptation and the outcomes of these adaptations. Additional research is also needed to explore the impacts of misalignments and adaptations and to determine what constitutes a successful outcome. Furthermore, this study took place in one location. Although we observed a variety of teams and ward areas to account for differences between different in-patient settings, this study should be repeated, and framework tested, in additional settings and health systems.

## Conclusions

Healthcare teams face frequent misalignments between demand and capacity and respond by adapting so that work can be successfully completed. Understanding the types of misalignments that occur and how teams adapt is important for improving systems of care. This study provides deeper insight into everyday clinical work on hospital wards and specifies the types of misalignments that occur and the mechanisms that are used to flexibly adapt, thus expanding knowledge of adaptive capacity and resilience in hospital teams. For all misalignment types observed across the five in-patient settings, *process adaptations* were the most frequently used adaptations. The exception to this was for *staffing misalignments*, which were most frequently responded to with *extra-role performance adaptations*. Of the three *process adaptations*, hospital workers most often adapted by changing *how* the process was done. These findings were consistent across all teams and settings and for all misalignment types. Adaptations, especially those that lead to longer-term, culturally accepted, informal practices have implications for patient care, healthcare quality and safety, burnout, staff retention, and outcomes. Many of these adaptations may have been invisible using traditional quality improvement methods, highlighting the importance of studying everyday work as it occurs. It is also important to understand how local adaptations affect downstream processes occurring at different spatial and temporal scales and further research is needed to understand these impacts of adaptations. The CARE model 2.0 specifies the types of misalignments and adaptations observed in hospital teams and thus provides further guidance for understanding adaptive capacity and how it is affected in practice. This understanding can be used to guide future research, to ground quality improvement efforts, and to contribute to system redesign on multiple organisational levels.

## Data Availability

The datasets used and/or analysed during the current study are available from the corresponding author on reasonable request.
